# Interaction Between Thyroid Hormones and Bone Morphogenetic Proteins in the Regulation of Steroidogenesis by Granulosa Cells

**DOI:** 10.3390/ijms26189127

**Published:** 2025-09-18

**Authors:** Kanon Motohashi, Yoshiaki Soejima, Koichiro Yamamoto, Nahoko Iwata, Atsuhito Suyama, Yasuhiro Nakano, Fumio Otsuka

**Affiliations:** Department of General Medicine, Okayama University Graduate School of Medicine, Dentistry and Pharmaceutical Sciences, 2-5-1 Shikata-cho, Kitaku, Okayama 700-8558, Japan; p7ca2xvh@s.okayama-u.ac.jp (K.M.); p32v0ja8@s.okayama-u.ac.jp (Y.S.); p5db54gf@s.okayama-u.ac.jp (A.S.); y-nakano@okayama-u.ac.jp (Y.N.)

**Keywords:** bone morphogenetic protein (BMP), thyroid hormone, steroidogenesis, ovary

## Abstract

Thyroid hormones are fundamental regulators of cellular differentiation, development, and metabolism. Their receptors are expressed in reproductive tissues, including the ovary, and dysregulation of thyroid hormone homeostasis has been associated with menstrual disturbances, infertility, and adverse pregnancy outcomes. Bone morphogenetic protein (BMP) ligands and their receptors are functionally involved in gonadotropin-induced ovarian steroidogenesis in an autocrine or paracrine manner. In this study, we examined the effects of thyroid hormones on steroidogenesis and their interplay with BMP signaling by using human granulosa-like KGN cells and primary rat granulosa cells (GCs). In KGN cells, triiodothyronine (T3) enhanced forskolin-induced expression of key steroidogenic enzymes involved in both estradiol biosynthesis and progesterone synthesis/metabolism, whereas thyroxine (T4) exerted minimal effects. In rat GCs, T3 treatment increased follicle-stimulating hormone (FSH)-stimulated estradiol production without altering progesterone output. T3 pretreatment attenuated BMP-6-induced phosphorylation of Smad1/5/9 in KGN cells, accompanied by upregulation of inhibitory Smad6 and downregulation of the BMP type II receptor. Conversely, BMP-6 stimulation elevated thyroid hormone receptor β expression, indicating reciprocal regulatory interactions between thyroid hormone and BMP pathways. Collectively, these findings suggest that thyroid hormones modulate steroidogenesis, at least in part, through suppression of endogenous BMP-6 signaling in granulosa cells.

## 1. Introduction

The hypothalamic–pituitary–thyroid axis is a feedback regulatory system that is essential for normal development, growth, neurodifferentiation, and metabolic regulation. This axis is activated in response to energy-sensing molecules, such as leptin and neuropeptide Y, which stimulate the hypothalamus to release thyrotropin-releasing hormone (TRH). TRH then acts on the anterior pituitary to promote the secretion of thyroid-stimulating hormone (TSH). TSH subsequently stimulates the thyroid gland to release triiodothyronine (T3) and thyroxine (T4), the two major thyroid hormones. Once secreted, T4 is converted in peripheral tissues by deiodinase (DIO) enzymes into either the more biologically active form, T3, or the biologically inactive form, reverse T3 (rT3) [1]. T3 mainly exerts its physiological effects by binding to thyroid hormone receptors (TRs) in target cells, with 10- to 30-fold higher affinity than T4 [2,3], thereby regulating the transcription of specific genes involved in metabolism, growth, and development.

Among various physiological systems, thyroid hormones play crucial roles in mammalian reproductive function. Proper ovarian function and successful pregnancy require not only an appropriately regulated immune system but also a well-balanced endocrine environment, including adequate levels of thyroid hormones [4]. Thyroid hormones are involved in the regulation of multiple levels of the hypothalamic–pituitary–gonadal axis, from upstream metabolic signaling to downstream processes governing sex steroid secretion and transport. In the reproductive system, thyroid hormones directly influence the uterus, placenta, and ovaries through TRs expressed in these tissues [1]. In the ovary, the expression of TRs in human oocytes, cumulus cells, and granulosa cells (GCs) has been revealed [5]. Notably, both T3 and T4 have been found in human follicular fluid [5,6]. These results support the hypothesis that thyroid hormones have direct roles in ovarian function. Research indicates that T3 potentiates the survival action of follicle-stimulating hormone (FSH) by inhibiting apoptosis and prompting proliferation of rat GCs [7] and that T3 promotes FSH-induced preantral follicular growth by upregulating FSH receptor, depending on oocyte-derived growth differentiation factor (GDF)-9 [8]. However, the detailed mechanism of the functions of thyroid hormones in the ovary remains unclear.

Thyroid disorders are well recognized for their close association with reproductive dysfunction in women of reproductive age. Hypothyroidism is commonly associated with ovulatory dysfunction due to some underlying mechanisms [4]. Elevated TRH levels can lead to hyperprolactinemia, which, along with altered pulsatile secretion of gonadotropin-releasing hormone (GnRH), contributes to a delayed luteinizing hormone (LH) surge and insufficient corpus luteum function. Another mechanism involves the reduction in sex hormone-binding globulin (SHBG) synthesis, thereby altering the peripheral metabolism of estradiol. By contrast, hyperthyroidism increases the production of SHBG, conversion of androgens to estrogen, and response of gonadotropin to GnRH [4]. TSH has also been reported to affect ovarian steroidogenesis and granulosa cell function [9,10]. Importantly, in addition to these indirect effects, accumulating evidence indicates that thyroid hormones directly modulate steroid production [11,12]. Thus, elucidating the direct impact of thyroid hormones on ovarian function is critical for a comprehensive understanding of the pathophysiological connections between thyroid dysfunction and reproductive health in clinical settings.

Bone morphogenetic proteins (BMPs), members of the transforming growth factor-β superfamily, have been shown to play pivotal roles in various endocrine organs as autocrine/paracrine factors [13]. In the ovary, various BMP ligands and receptors expressed in the follicles with cell-specific patterns regulate multiple biological processes including cell proliferation, apoptosis, differentiation, and morphogenesis during folliculogenesis, ovulation, and luteogenesis [14]. Binding of BMP ligands to their cognate receptors, including type I and type II subtypes, induces activation of Smad signaling [15]. Phosphorylated Smad1/5/9 proteins form a complex with Smad4, which enters the nucleus and drives target gene transcription. These processes are inhibited by BMP-binding proteins and inhibitory Smads (Smad6/7). Regarding the functional roles of the ovarian BMP system, BMP ligands exhibit partially overlapping activities, although each ligand also plays a distinctive role in follicular development. Among the BMPs, BMP-6 has been reported to be expressed in the oocytes and GCs of healthy Graafian follicles and to inhibit FSH-induced progesterone synthesis in GCs [16,17,18]. BMP-6 inhibits forskolin (FSK)- but not cAMP-induced progesterone production and also decreases follicle-stimulating hormone (FSH)- and FSK-stimulated cAMP synthesis, suggesting that BMP-6 suppresses FSH action through the inhibition of adenylate cyclase activity [17]. Since BMP-6 expression in granulosa cells declines rapidly during dominant follicle selection [16], BMP-6 likely plays an important role in this process. Notably, we have recently demonstrated that BMP-6 modulates steroidogenesis by interacting with various factors, including growth hormone, androgen, aldosterone, melatonin, incretins, and orexins in granulosa cells [19,20,21,22,23,24]. However, the potential functional interaction between thyroid hormones and ovarian BMPs remains to be elucidated.

Accordingly, the present study aimed to elucidate the direct effects of thyroid hormones on steroidogenesis, with a particular focus on their interaction with the BMP system in GCs.

## 2. Results

**Figure 1:**
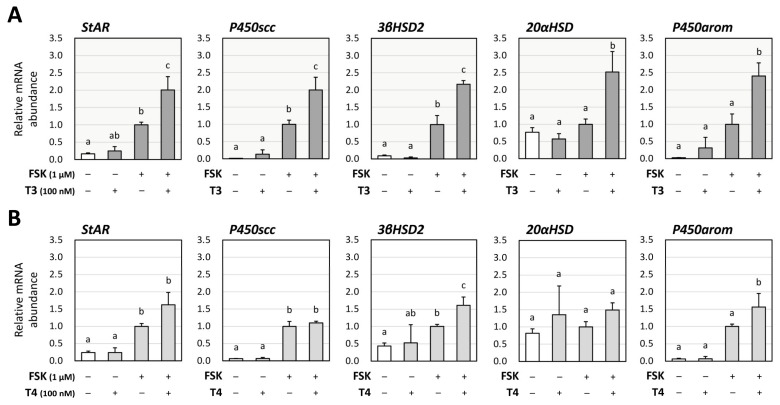
**Effects of thyroid hormones on the expression of steroidogenic enzymes in human granulosa KGN cells.** KGN cells were treated with (**A**) T3 or (**B**) T4 (100 nM each) in the presence and absence of FSK (1 μM) for 24 h. Total RNAs were extracted, and the mRNA levels of steroidogenic enzymes (StAR, P450scc, 3βHSD2, 20αHSD, and P450arom) were determined using RT-qPCR. The expression levels were standardized by RPL19 and expressed as fold changes. Data are shown as means ± SEM and were statistically analyzed using ANOVA followed by Tukey’s post hoc test. Values with different superscript letters are significantly different at *p* < 0.05.

To evaluate the effects of thyroid hormones (T3 and T4) on steroid production by GCs, their impact on the expression of key steroidogenic enzymes were examined using human KGN cells. As KGN cells have been reported to have low levels of FSH receptors [25], FSK was used to evaluate steroidogenesis. Real-time quantitative PCR (RT-qPCR) was performed to access the relative mRNA abundance of steroidogenic enzymes, steroidogenic acute regulatory protein (StAR), steroid side-chain cleavage enzyme (P450scc), 3β-hydroxysteroid dehydrogenase 2 (3βHSD2), 20α-hydroxysteroid dehydrogenase (20αHSD), and aromatase (P450arom) in KGN cells. As shown in Figure 1, in the absence of FSK (1 μM), treatment with T3 (100 nM) or T4 (100 nM) alone did not change the mRNA levels of the enzymes. Of note, treatment with T3 significantly increased FSK-induced mRNA levels of StAR, P450scc, 3βHSD2, 20αHSD, and P450arom (Figure 1A). On the contrary, as shown in Figure 1B, treatment with T4 for 24 h increased FSK-induced mRNA levels of 3βHSD2 and P450arom, but not that of StAR, P450scc, and 20αHSD.

**Figure 2:**
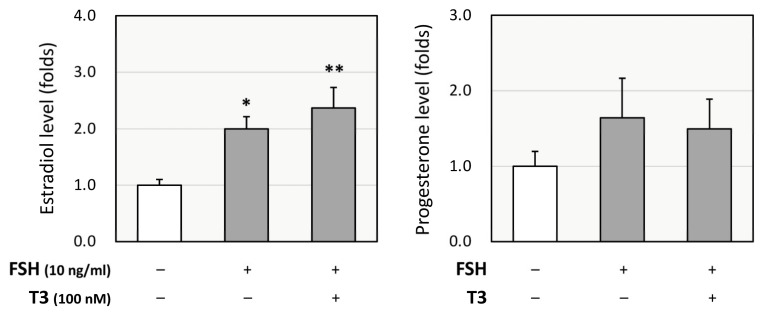
**Effects of T3 on steroidogenesis by rat granulosa cells (GCs).** Rat primary GCs were treated with FSH (10 ng/mL) with or without T3 (100 nM) for 48 h. The conditioned media were collected, and the concentrations of estradiol and progesterone were quantified using ELISA and expressed as fold changes. Results are shown as means ± SEM and were statistically analyzed using ANOVA followed by Tukey’s post hoc test; *, *p* < 0.05; **, *p* < 0.01 between the indicated groups.

As T3 has been shown to have a greater impact on the steroidogenic pathway than T4, we focused on the effects of T3 in GCs. Rat primary GCs were cultured with FSH (10 ng/mL) with or without T3 (100 nM) for 48 h, and the culture supernatant was tested for enzyme-linked immunosorbent assay (ELISA). T3 treatment enhanced FSH-induced production of estradiol but not progesterone (Figure 2). According to these results, it was hypothesized that T3 treatment increases FSH-induced estradiol production by upregulating P450arom and that it does not affect progesterone production due to the counteracting effects of progesterone-synthesizing enzymes (StAR, P450scc, and 3βHSD2) and progesterone-degrading enzyme (20αHSD).

**Figure 3:**
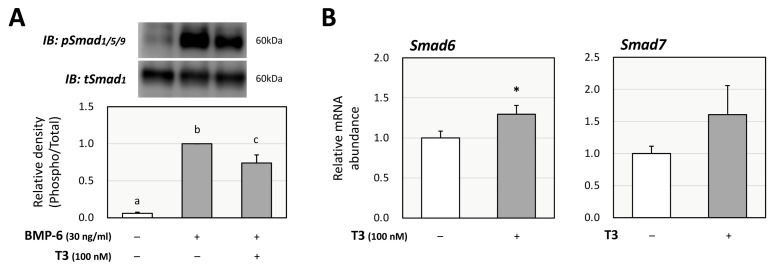
**Effects of T3 on Smad signaling induced by BMP-6 in KGN cells.** (**A**) KGN cells were pretreated with T3 (100 nM) for 24 h and then stimulated with BMP-6 (30 ng/mL) for 1 h. The cell lysates were analyzed by SDS-PAGE/immunoblotting using antibodies against pSmad1/5/9 and tSmad1. The signal intensities of pSmad1/5/9 were normalized to those of tSmad1. The provided data are representative of at least three independent experiments and are expressed as fold changes. (**B**) KGN cells were treated with T3 (100 nM) for 24 h. Total RNAs were extracted, and the mRNA levels of inhibitory Smads (Smad6 and Smad7) were standardized by RPL19 mRNA levels and expressed as fold changes. Results are shown as means ± SEM and were statistically analyzed using ANOVA followed by Tukey’s post hoc test (**A**) or the unpaired *t*-test (**B**). Values with different superscript letters are significantly different at *p* < 0.05 (**A**); *, *p* < 0.05 vs. control groups (**B**).

Next, the effects of T3 on BMP-6 signaling were evaluated using KGN cells, as we have reported that BMP-6 affects FSH-induced steroid production by GCs [25]. BMP signaling activity was evaluated under FSK-free conditions to assess the direct effect of T3 on BMP signaling. As shown in Figure 3A, Western blot analysis showed that the phosphorylation of Smad1/5/9 induced by BMP-6 (30 ng/mL) for 1 h was significantly reduced by T3 (100 nM) pretreatment for 24 h. To identify the mechanism by which the pretreatment with T3 inhibits BMP-6 signaling, the effects of T3 on inhibitory Smads were examined. Treatment with T3 (100 nM) increased the mRNA levels of Smad6, but not those of Smad7, with statistical significance (Figure 3B).

**Figure 4:**
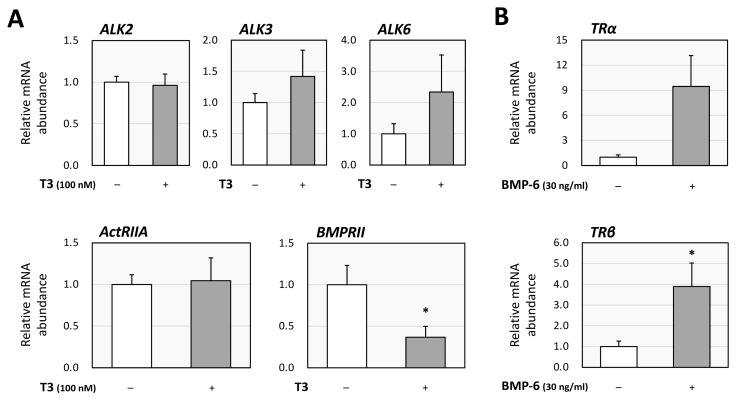
**Interactions between T3 and BMP signaling in KGN cells.** (**A**) KGN cells were treated with T3 (100 nM) for 24 h. Total cellular RNAs were extracted, and subsequently the mRNA levels of BMP receptors (ALK-2, -3, -6, ActRIIA, and BMPRII) were standardized by RPL19 mRNA levels and expressed as fold changes. (**B**) KGN cells were treated with BMP-6 (30 ng/mL) for 24 h. Total cellular RNAs were extracted, and the mRNA levels of thyroid hormone receptors (TRα and TRβ) were standardized by RPL19 mRNA levels and expressed as fold changes. Results are shown as means ± SEM and were statistically analyzed using the unpaired *t*-test; *, *p* < 0.05 vs. control groups.

Then, the functional interaction between the effects of T3 and BMP-6 was further investigated from the perspective of the impact on each receptor in KGN cells. Treatment with T3 (100 nM) downregulated the expression of BMP type-II receptor (BMPRII), but not that of activin receptor-like kinases (ALK-2, ALK-3, and ALK-6) or activin A receptor type 2 (ActRIIA; Figure 4A). These results suggest that T3 regulates steroid production by inhibiting BMP-6 signaling through upregulation of Smad6 and downregulation of BMPRII in ovarian GCs. Regarding the impact on the expression of TRs, it was shown that treatment with BMP-6 (30 ng/mL) upregulated the expression of thyroid hormone receptor β (TRβ), but not that of thyroid hormone receptor α (TRα), with statistical significance (Figure 4B).

## 3. Discussion

**Figure 5:**
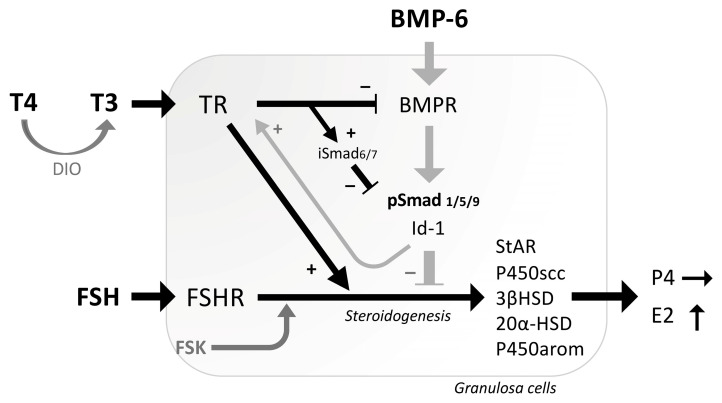
**Functional interaction between thyroid hormones and BMP-6 in the steroidogenesis by GCs.** T4 is converted by deiodinase (DIO) enzymes into the more biologically active form T3. T3 enhances FSH/FSK-induced expression of estradiol-synthesizing enzyme (P450arom), as well as progesterone-synthesizing enzymes (StAR, P450scc, 3βHSD2) and progesterone-metabolizing enzyme (20αHSD), leading to the enhanced production of estradiol (E2) but not progesterone (P4). T3 suppresses BMP-6 signaling by reducing BMP-6-induced Smad1/5/9 phosphorylation through upregulation of inhibitory Smad (iSmad) and downregulation of BMPR. On the contrary, BMP-6 upregulates the expression of thyroid hormone receptor (TR). The functional interrelationships between the activities of thyroid hormones and BMP-6 for steroidogenesis were demonstrated in GCs.

In the present study, the interactions between thyroid hormones and the BMP signaling on steroidogenesis were revealed in human and rat GCs (Figure 5). T3 actions enhanced FSK-induced expression of estradiol-synthesizing enzyme, as well as progesterone-synthesizing and progesterone-metabolizing enzymes, whereas T4 had only slight effects on expression of steroidogenic enzymes by human GCs. Consistent with the results, T3 treatments enhanced FSH-induced estradiol production but not progesterone. The presence of T3 also inhibited BMP-6-induced Smad1/5/9 phosphorylation by upregulating inhibitory Smad6 and downregulating BMPRII. Notably, BMP-6 enhanced the expression of TRβ in GCs. These findings suggest that thyroid hormones, particularly T3, regulate steroidogenesis both directly and by inhibiting the BMP signaling and that BMP-6 upregulates the expression of TRs, leading to the fine-tuning of steroid production by GCs.

Among thyroid hormones, T3 was found in the present study to enhance FSH-induced estradiol production by GCs. Consistently, a previous study reported that T3 increased estradiol secretion in human chorionic gonadotropin (hCG)-stimulated porcine GCs [26]. By contrast, experiments using human luteinized GCs showed that T3 and T4 (100 nM each) upregulated the expression of StAR while reducing that of P450arom [27], and that T3 reduced hCG-induced secretion of progesterone in a dose-dependent manner [12]. Meanwhile, in granulosa and theca co-culture, which resembles follicles in vivo, decreased secretion of estradiol in response to T3 was observed in porcine cells [26]. It was also reported that T4 slightly increased production of estradiol and progesterone in human granulosa cells from preovulatory follicles [28]. In mouse granulosa cells, however, T3 increased FSH-induced secretion of estradiol and progesterone [29]. These results indicate that thyroid hormones have multifaceted and phase/species-dependent effects on ovarian function, which may account for the association between thyroid function and reproductivity.

Our results indicated that T3 has stronger actions for steroid synthesis in the ovary than T4. This effect may be explained by the higher affinity of T3 for TRs than T4 [2,3]. T4 is converted to the more biologically active form T3 or the biologically inactive form rT3 by deiodinase type 1 (DIO1). Deiodinase type 2 (DIO2) exclusively converts T4 to T3, although deiodinase type 3 (DIO3) inactivates thyroid hormones by converting T4 to rT3 [30]. Human ovarian tissue reportedly expresses DIO2 and DIO3, but not DIO1, which indicates its capacity to generating T3 in the presence of T4 [31,32]. Moreover, the actions of thyroid hormones require the presence of thyroid hormone transporters to facilitate their cellular uptake and efflux. Several transporters, including monocarboxylate transporter 8 (MCT8), organic anion transporting polypeptide 1C1 (OATP1C1), large neutral amino acid transporter 1 (LAT1) and 2 (LAT2), and the escort protein 4F2hc, have been reported to be expressed in the mammalian ovary [33,34]. These results suggest that thyroid hormones are taken up into the cells and converted to bioactive T3 by DIO2, which modulates steroid synthesis in the ovary.

The present results also confirmed that T3 regulates steroid production by modulating the BMP signaling through inhibitory Smads and BMPRs in GCs. Our previous studies have demonstrated that various hormones and factors, including growth hormone, insulin-like growth factor-I, androgen, aldosterone, melatonin, orexin A, prolactin, somatostatins, incretins, oxytocin, and vasopressin, regulate the expression of inhibitory Smads as a key negative feedback factor in steroidogenesis [35]. In this study, T3 inhibits the BMP signaling by upregulating Smad6 expression, which indicates that thyroid hormones act as an endogenous regulator for the BMP system in GCs. Furthermore, in human luteinized GCs, T3 has been reported to enhance hCG-induced cyclic adenosine monophosphate (cAMP) synthesis [12], whereas both T3 and T4 modulates AKT phosphorylation [27,29]. These results indicate that thyroid hormones modulate ovarian steroidogenesis by interacting with various intracellular signaling pathways, including the BMP signaling and cAMP/protein kinase A (PKA) pathway.

From a clinical perspective, thyroid disease is closely related to female reproduction. In particular, the prevalence of the autoimmune thyroid disease (AITD), including Graves’ disease and Hashimoto’s thyroiditis, as well as endometriosis and polycystic ovary syndrome (PCOS), is significantly higher in infertile women compared to age-matched fertile women [36]. A large cross-sectional survey demonstrated that women with endometriosis had a significantly higher frequency of autoimmune diseases, including hypothyroidism [37]. Patients with PCOS reportedly exhibit a higher prevalence of anti-thyroglobulin or anti-thyroperoxidase antibodies compared to individuals without PCOS [38,39]. Moreover, enhanced expression of BMP-6 in GCs [40,41] and reduced expression of BMP-15 in follicular fluid [42] have been observed in patients with PCOS. Taken together, these findings suggest a strong correlation among thyroid disease, abnormalities in the BMP system, and ovarian dysfunction.

In conclusion, the present findings demonstrated that T3, among thyroid hormones, enhances gonadotropin-stimulated estradiol synthesis in GCs. This regulation is mediated through a bidirectional interaction between T3 and BMP-6, involving inhibitory Smads, BMPRs, and TRs. Elucidating the functional crosstalk between thyroid hormones and the BMP pathway in GCs could advance our understanding of the molecular mechanisms underlying ovarian dysfunction and infertility linked to thyroid dysregulation.

## 4. Materials and Methods

### 4.1. Experimental Reagents

Culture media for KGN cells consisted of a 1:1 mixture of Dulbecco’s Modified Eagle’s Medium and Ham’s F-12 medium (DMEM/F12) supplemented with HEPES buffer, while media for rat granulosa cells (GCs) comprised McCoy’s 5A medium and Medium 199 (Thermo Fisher Scientific, Waltham, MA, USA). Fetal bovine serum (FBS), penicillin–streptomycin, 3-isobutyl-1-methylxanthine (IBMX), diethylstilbestrol (DES), 4-androstene-3,17-dione, ovine follicle-stimulating hormone (FSH), and forskolin (FSK) were purchased from Sigma-Aldrich (St. Louis, MO, USA). Recombinant human BMP-6 was obtained from R&D Systems (Minneapolis, MN, USA), and 3,3′,5′-triiodo-L-thyronine (T3) and L-thyroxine (T4) sodium salt were obtained from FUJIFILM Wako Pure Chemical Industries (Osaka, Japan).

### 4.2. Preparations of Human and Rat Granulosa Cells

KGN cells, derived from human GC tumors [43], were cultured in DMEM/F12 supplemented with 10% FBS and penicillin–streptomycin in a humidified atmosphere of 5% CO_2_ at 37 °C. Rat GCs were obtained from female Sprague–Dawley rats (Charles River, Wilmington, MA, USA). To induce follicular development, 20-day-old female rats were subcutaneously implanted with Silastic tubes containing DES (10 mg). After 3 days, ovaries were excised, and GCs were collected by puncturing follicles with a 27-gauge needle. The resulting cell suspension in Medium 199 was passed through nylon meshes with pore sizes of 100 and 40 μm (BD Falcon, Bedford, MA, USA) to remove oocytes, as previously described [35,44]. Isolated GCs were cultured in serum-free McCoy’s 5A medium supplemented with penicillin–streptomycin under a humidified 5% CO_2_ atmosphere at 37 °C. All animal procedures were approved by the Institutional Animal Care and Use Committee of Okayama University.

### 4.3. Real-Time Quantitative PCR Analysis

KGN cells (1 × 10^5^ cells/mL) were cultured in serum-free DMEM/F12 in 12-well plates with FSK (1 μM), T3 (100 nM), T4 (100 nM), BMP-6 (30 ng/mL), or their combinations. The concentrations of FSK and BMP-6 were selected based on our previous studies [22,45], while those of T3 and T4 were based on preliminary experiments, which showed that the changes in the expression of steroidogenic enzymes were dose-dependent, and that 100 nM of T3 and T4 produced sufficient responses. After 24 h culture, total RNA was extracted using TRI Reagent^®^ (Cosmo Bio Co., Ltd., Tokyo, Japan), and RNA yield was measured with a NanoDrop™ One spectrophotometer (Thermo Fisher Scientific, Waltham, MA, USA). Reverse transcription (RT) was performed with 1 μg of total RNA using ReverTra Ace^®^ (TOYOBO Co., Ltd., Osaka, Japan) and random primers with deoxynucleotide triphosphates. Real-time quantitative PCR was conducted on a MyGo Pro qPCR Instrument (IT-IS Life Science Ltd., Dublin, Ireland). The primer sets used in this study were shown in Table 1 [45]. To avoid amplification of genomic DNA, primers were designed to span different exons of the target genes. mRNA levels were quantified using the comparative threshold cycle (Ct) method, with RPL19 serving as the internal control. Relative expression levels were calculated using the 2^−ΔΔCt^ method. The results are shown as ratios of target mRNA levels to RPL19 mRNA levels.

### 4.4. Assays for Estradiol and Progesterone

Rat primary GCs (1 × 10^5^ cells in 200 μL) were cultured under serum-free conditions in McCoy’s 5A medium supplemented with 4-androstene-3,17-dione (100 nM) in 96-well plates. Cells were exposed to FSH (10 ng/mL), with or without T3 (100 nM), for 48 h. The FSH concentration was selected based on previously published protocols from our group [35,46]. Estradiol and progesterone concentrations in the culture supernatants were quantified using commercially available ELISA kits (Cayman Chemical Co., Ann Arbor, MI, USA). In cell-free medium, estradiol and progesterone concentrations were below the respective limits of detection (<15 and <10 pg/mL, respectively).

### 4.5. Western Immunoblotting Analysis

KGN cells (1 × 10^5^ cells/mL) were cultured in serum-free DMEM/F12 supplemented with T3 (100 nM) for 24 h, followed by stimulation with BMP-6 (30 ng/mL) for 1 h in 12-well plates. After treatment, cells were lysed in 100 μL of RIPA buffer (Upstate Biotechnology, Lake Placid, NY, USA) containing 1 mM Na_3_VO_4_, 1 mM NaF, 2% sodium dodecyl sulfate (SDS), and 4% β-mercaptoethanol. Lysates were subjected to SDS–polyacrylamide gel electrophoresis (SDS–PAGE) and immunoblotting using antibodies against phosphorylated Smad1/5/9 (pSmad1/5/9) and total Smad1 (tSmad1; Cell Signaling Technology, Beverly, MA, USA). Band intensities were visualized and quantified using the C-DiGit^®^ Blot Scanner System (LI-COR Biosciences, Lincoln, NE, USA). Smad1/5/9 phosphorylation was evaluated by calculating the ratio of pSmad1/5/9 to tSmad1 signal intensities.

### 4.6. Statistics

Data are expressed as the mean ± standard error of the mean (SEM) from at least three independent experiments, each performed in triplicate. Statistical analyses were conducted using ANOVA followed by Tukey’s post hoc test or an unpaired *t*-test, as appropriate. A *p*-value of <0.05 was considered to indicate statistical significance.

## Figures and Tables

**Table 1:** Primer sets for the real-time quantitative PCR analysis (human).

Gene	Sense Primer (5′ to 3′)	Antisense Primer (5′ to 3′)	Product (bp)	Accession No.
RPL19	ATCGATCGCCACATGTATCA	CGTGCTTCCTTGGTCTTAGA	167	NM_000981
StAR	GGCTACTCAGCATCGACCTC	CATCCCACTGTCACCAGATG	250	NM_000349
P450scc	GGAAATTACTCGGGGGACAT	CACATGGTCCTTCCAGGTCT	228	NM_000781
3βHSD2	CCACACCGCCTGTATCATTG	TCCAGAGGCTCTTCTTCGTG	204	NM_000198
20αHSD	GATCCCACCGAGAAGAACCA	TCAAACACCTGCACGTTCTG	196	AB031083.1
P450arom	CAGAGGCCAAGAGTTTGAGG	ACACTAGCAGGTGGGTTTGG	223	NM_000103
Smad6	TGCAACCCCTACCACTTCAGC	TTCACCCGGAGCAGTGATGAG	165	NM_005585
Smad7	TGTGCAAAGTGTTCAGGTGGC	GGGTATCTGGAGTAAGGAGG	169	NM_001190821
ALK2	TTCCTCACTGAGCATCAACG	TAATGAGGCCAACCTCCAAG	221	NM_001105
ALK3	TTTATGGCACCCAAGGAAAG	TGGTATTCAAGGGCACATCA	156	NM_001406559
ALK6	GCCCATCGAGATCTGAAAAG	TAGCAACCTCCCAAAGGATG	250	NM_001203
ActRIIA	AAAAGATGGCCACAAACCTG	CCAACCTGTCCATGGGTATC	153	NM_001278579
BMPRII	GACAACATTGCCCGCTTTAT	ATCTCGATGGGAAATTGCAG	237	NM_001204
TRα	TAGTCTCCGACGCCATCTTT	CAGAAGTGCGGAATGTTGTG	211	NM_199334.5
TRβ	GCAGCACGTTGAAAAATGAA	GTGGCTTTGTCACCACACAC	208	NM_000461.5

RPL19, ribosomal protein L19; StAR, steroidogenic acute regulatory protein; P450scc, steroid side-chain cleavage enzyme; 3βHSD2, 3β-hydroxysteroid dehydrogenase 2; 20αHSD, 20α-hydroxysteroid dehydrogenase; P450arom, aromatase; ALK, activin receptor-like kinase; ActRIIA, activin A receptor type 2A; BMPRII, BMP type-II receptor; TR, thyroid hormone receptor; bp: base pairs.

## Data Availability

The data presented in this study are available on request from the corresponding author.

## Abbreviations

The following abbreviations are used in this manuscript:

ActRIIA	activin A receptor type 2A
ALK	activin receptor-like kinase
20αHSD	20α-hydroxysteroid dehydrogenase
3βHSD2	3β-hydroxysteroid dehydrogenase 2
BMP	bone morphogenetic protein
BMPRII	BMP type-II receptor
DIO	deiodinase
FSH	follicle-stimulating hormone
FSK	forskolin
P450arom	aromatase
P450scc	steroid side-chain cleavage enzyme
RPL19	ribosomal protein L19
StAR	steroidogenic acute regulatory protein
TR	thyroid hormone receptor
T3	triiodothyronine
T4	thyroxine
